# What the National Insurance Commissioners Cannot Do

**Published:** 1912-11-30

**Authors:** 


### What the National Insurance Commissioners Cannot Do.

NO EXEMPTIONS AS SUCH.

A KNOWLEDGEABLE CORRESPONDENT writes: May I draw your attention to a note in last week's THE HOSPITAL which is likely to mislead? The Insurance Commissioners have not "exempted" hospital doctors from insurance under the Act: they have no power to exempt anyone, except in a few cases where a man or woman is engaged in a subsidiary employment, as specified in a special order. All that they have done is to declare in their judicial capacity under Section 66 of the Act that in their opinion hospital doctors are not employed under a contract of service at all, and therefore it follows that the Act has no application to them. The decision is no doubt important to the doctors concerned, but it is of no importance to other hospital workers; and as for drawing any inference from it with regard to nurses, it may be pointed out that the Commissioners (also under Section 66) have decided that nurses *are* employed under a contract of service, that the Act therefore applies to them, and they must be insured. This idea about "exemption" seems a very common error. If a person *is* employed under a contract of service, he or she has got to be insured, and the Commissioners must, of course, see that they are insured; they cannot exempt people who do not want the Act to apply to them. An amending Act is also quite out of the question, at least during the next twelve months, and nurses would not be included in it in any event I am sure.

